# Everyday storage and handling of PET bottled water increase human exposure to nano- and microplastics: Influence of socio-economic factors

**DOI:** 10.1016/j.watres.2026.125569

**Published:** 2026-02-14

**Authors:** Kazi Albab Hussain, Haoyu Dong, Alex Tamayo-Aguilar, Seulki Kim, Cesar Augusto Gomez Pena, Silvia Foss, Lucia Fernandez-Ballester, Yongfeng Lu, Yusong Li

**Affiliations:** a Department of Civil and Environmental Engineering, University of Nebraska-Lincoln, Lincoln, NE, 68588-053, United States; b Department of Electrical & Computer Engineering, University of Nebraska-Lincoln, Lincoln, NE, 68588-0511, United States; c Department of Mechanical & Materials Engineering, University of Nebraska-Lincoln, Lincoln, NE, 68588-0526, United States; d Department of Sociology, University of Nebraska-Lincoln, Lincoln, NE, 68588, United States; e Facultad de Ingeniería Civil, Universidad Tecnológica de Panamá, Avenida Tecnológica de Panamá, Panamá 0819-0728, Panamá; f University of Michigan, Ann Arbor, MI, 48109, United States

**Keywords:** Nanoplastics, Microplastics, Single-use pet water bottles, High-temperature exposure, Low-temperature exposure, Mechanical agitation

## Abstract

This study quantified the release of nanoplastics and microplastics (NMPs) from single-use polyethylene terephthalate (PET) water bottles under real-world storage and handling conditions and examined how socio-economic factors influence exposure behaviors. Eight leading U.S. bottled water brands were tested under high temperatures (60 °C), mechanical shaking (200 rpm), and 15-day temperature cycling to simulate storage in vehicles or outdoors. The highest release occurred under combined heat and shaking, where nanoparticle concentrations increased by 9.29-fold and microparticles also rose significantly. Prolonged freeze - thaw and high temperature cycling also significantly elevated nanoparticle concentrations, though microparticle release was less consistent. Raman spectroscopy identified PET, polyethylene, and polypropylene particles originating from both bottle body and caps. Surface degradation, rather than bulk changes, was the likely driver of particle release, as supported by differential scanning calorimetry showing heating-induced aging without increased release. A statewide survey (*n* = 1673) in Nebraska revealed that individuals with higher awareness of microplastics and higher education levels were less likely to consume bottled water or store it under heat, underscoring the role of knowledge and behavior in shaping exposure risks.

## GRAPHICAL ABSTRACT

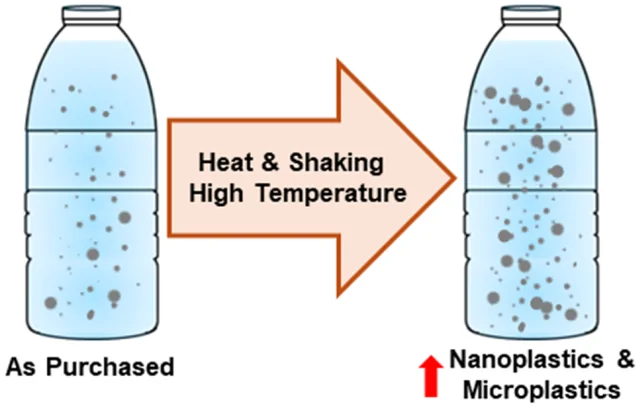

## 1. Introduction

The global bottled water market is projected to reach a consumption of 460 billion liters by 2030, highlighting a growing dependence on single-use plastic bottles (Dickie, 2023). The presence of nanoplastic and microplastics (NMPs) in bottled water has been widely documented (Gambino et al., 2022; Huan et al., 2023; Kankanige and Babel 2020; Li et al., 2022; Muhib et al., 2022; Oßmann et al., 2018; Praveena et al., 2022, Tse et al. 2022, Weisser et al., 2021, Zhou et al., 2021) across regions, including the USA, Iran, Thailand, Chile, Italy, Germany, and China (Almaiman et al., 2021; Kankanige and Babel 2020; Makhdoumi et al., 2021; Mason et al., 2018; Nacaratte et al., 2023; Oßmann et al., 2018; Parveen et al., 2022; Qian et al., 2024; Samandra et al., 2022; Schymanski et al., 2018; Tse et al., 2022; Winkler et al., 2019; Zhou et al., 2021; Zuccarello et al., 2019). Early studies (Kankanige and Babel 2020; Makhdoumi et al., 2021; Schymanski et al., 2018) primarily focused on microplastics larger than 5 μm, but more recent work (Qian et al., 2024) revealed nanoscale particles (200–1000 nm) at much higher concentrations, reaching 2.4 ± 1.3 × 10^5^ particles per liter.

Importantly, human exposure to NMPs from bottled water depends not only on the presence of particles in the product, but also on how bottled water is stored, handled, and consumed in everyday life. Bottled water consumption patterns vary widely across populations and are influenced by trust in municipal water quality, access to infrastructure, income, education, and awareness of health risks (Onufrak et al., 2014; Rosinger et al., 2018). Individuals with limited access to safe tap water or lower trust in public supplies may rely more heavily on bottled water, potentially increasing cumulative exposure. At the same time, storage and handling behaviors—such as leaving bottled water in vehicles or outdoor environments—may further modify exposure by influencing particle release from plastic packaging.

Only a limited number of studies have examined how realistic consumer-use conditions affect the release of plastic particles from single-use PET bottles. Mechanical stresses—such as repeated cap opening (Winkler et al., 2019), abrasion at the bottleneck (Singh, 2021), or twisting (Sobhani et al., 2020)—can generate microplastics, but these represent only a subset of real-world conditions. For instance, bottled water is often left or stored in personal vehicles, garages, or outdoors for extended periods during both summer and winter, where it may be subjected to elevated temperatures, freezing, and mechanical agitation. According to the National Oceanic and Atmospheric Administration (NOAA), the interior of a car can reach 82 °C to over 93 °C on a hot summer day. Additionally, driving generates mechanical vibrations ranging from 100 to 300 rpm, while winter storage in unheated garages or outdoor areas may expose bottles to subfreezing temperatures. Despite the prevalence of these conditions, their impact on NMP release from single-use PET bottles remains largely unexplored.

Critically, the likelihood of bottled water being exposed to such high-risk conditions is not uniform across consumers. Storage practices, transportation habits, and consumption frequency differ by socioeconomic context, lifestyle, and awareness. Global analyses have shown that plastic consumption is higher in high-income and industrialized regions (Hossain et al., 2021), but the role of socioeconomic and behavioral factors in shaping exposure-relevant practices, such as storage location and duration, remains insufficiently understood. Without integrating consumer behavior into experimental design, laboratory measurements of particle release cannot be readily translated into real-world exposure risk.

In this study, we address these critical gaps by combining laboratory simulations of real-world storage and transport scenarios with socio-behavioral data. We selected eight of the most popular single-use PET bottled water brands in the United States, collectively accounting for over 50 % of the still water market share. Experiments were designed to replicate extended storage of bottled water inside a car under both summer and winter conditions, and the resulting release of NMPs was quantified. In parallel, we surveyed Nebraska residents on their bottled water consumption habits, including consumption volume and storage behaviors, and analyzed how these behaviors correlate with household income, education level, and awareness of microplastics. Together, these approaches offer a holistic understanding of NMP exposure risk and its variability across consumer groups.

## 2. Materials and methods

### 2.1. Materials

Eight of the most popular single-use bottled-water brands in the USA were selected for this study. All bottles were 500 mL in volume and contained unflavored still water. All bottles were made of PET. Seven brands underwent various purification processes, of which four had minerals added and one included both mineral addition and pH adjustment; one brand contained natural spring water.

### 2.2. Release experiments.

To simulate common real-life scenarios where PET bottles are exposed to various conditions, a series of release experiments were conducted. All the experiments were performed in duplicate.

- **Baseline**: Bottled water was tested as purchased, and compared with bottles refilled deionized (DI) water (18.2 MΩ cm, Barnstead Nano-pure Systems, Thermo Scientific Inc., Waltham, MA).
- **High-Temperature**: Experiments were conducted at 60 °C for 12 h in an air convection oven, replicating the high temperatures that PET bottles may experience inside a car or outdoors during summer.
- **Cold Exposure**: To simulate winter conditions, PET bottles were placed in a freezer at −12 °C for 24 h.
- **Shaking Exposure**: To simulate the mechanical agitation PET bottles may experience in a moving car, experiments were conducted at 200 rpm for 12 h using a shaker at room temperature (25 °C). The setup represents typical car vibration frequencies, which range from 100 to 300 rpm.
- **High Temperature with Shaking:** To simulate the combined effects of high temperature and shaking, an experiment was conducted at 60 °C with shaking at 200 rpm for 12 h in an incubator shaker. This temperature was selected because several field studies have shown that the interior of vehicles can reach 40–75 °C depending on weather, sunlight intensity, and time of year (Grundstein et al., 2009; McLaren et al., 2005), making 60 °C an extreme yet realistic upper-bound exposure scenario. The shaking speed of 200 rpm (~3.3 Hz) falls within the 1–10 Hz vibration frequency range typically experienced by objects inside moving vehicles (Fan et al., 2018). In addition, prior experimental studies that simulated real-world transportation conditions have also used shaking speeds around 200 rpm (Strnad et al., 2025; Wang et al., 2024), supporting the relevance of this parameter for modeling transport-induced mechanical stress.
- **Temperature Cycling:** To simulate prolonged outdoor storage of bottled water during summer or winter, release experiments were conducted over 15 days with four brands of PET bottles, all of which initially exhibited low baseline concentrations of nano- and microparticles. Each day, bottles were exposed to either 60 °C (to mimic summer conditions) or freezing temperatures (to mimic winter), followed by 12 h at room temperature. These daily fluctuations represent two experimental conditions: high-temperature cycling and freeze–thaw cycling.

### 2.3. Effluent characterization.

Nanoparticle and microparticle quantification were conducted following the methodologies published by Hussain et al. (2023). For nanoparticle analysis, effluent samples were directly examined using Nanoparticle Tracking Analysis (NTA) with a NanoSight NS300 (Malvern Panalytical Ltd, UK) equipped with a 532 nm green laser. This system detects and counts particles ranging from 10 nm to 1 μm in diameter. Two replicate analyses were performed for each sample, and a control analysis was conducted for each brand of bottled water, where the control samples did not undergo any experimental conditions. Because NTA does not provide chemical identification, these baseline measurements were used to account for background nanoparticles originally present in the water and to contextualize concentration changes observed after the exposure conditions.

We used an EVOS FL Auto Imaging System (Thermo Fisher Scientific, Waltham, MA) at 40x magnification with 10x zoom for microparticle quantification. 20 μL of each effluent sample was placed on a clean glass slide, and 16 images were captured from the dried area using the imaging system. The images were processed with ImageJ (National Institutes of Health, USA) to analyze particle count and size using consistent background noise removal. Two replicates were performed per sample, totaling 32 images analyzed. Control samples were not exposed to experimental conditions and were analyzed following the same procedure. ImageJ was used solely for particle detection and sizing and did not attempt chemical or compositional classification of particles. To address the presence of non-plastic particles such as salt crystals, brand-specific baseline controls were processed using identical methods and used to account for background particulates and isolate particles released under the exposure conditions. The robustness of the method was further evaluated through salt spike–recovery experiments using 0.9 % NaCl in deionized water (Text S2). To facilitate the understanding of potential salt-related interference, conductivity values for each bottled water brand are reported in the Supporting Information (Table S1).

To identify the polymer type of the particles, Raman spectroscopy was employed using a Renishaw InVia Raman spectrometer equipped with a 514 nm laser and an optical microscope with approximately 1 μm resolution. This analysis was conducted on particles from control water samples and effluent water samples collected after exposure to 60 °C with shaking at 200 rpm for 12 h. Both sample types were initially filtered through a 25 mm diameter gold-coated track-etched membrane filter with a 0.8 μm pore size (Sigma-Aldrich Inc., MA). Raman spectra were recorded in the 500 to 4000 cm^−1^ range at 100 % laser power. A total of 40 spectra were collected for each sample, and the Raman spectral peaks were compared to standard microplastic Raman spectra to determine the polymer type.

Atomic Force Microscopy–Infrared Spectroscopy (AFM-IR) measurements were performed using an Anasys nanoIR2-s system operated in contact mode. Effluent collected after the release experiment was sequentially drop-cast onto a 30-nm gold-coated silicon substrate and dried under ambient laboratory conditions. A total of five droplets (10 μL each) were deposited to ensure adequate particle loading and promote a more homogeneous distribution of particles. For the release experiment, the single-use PET bottle was emptied and refilled with HPLC-grade water, and the experiment was conducted at 200 rpm and 60 °C for 12 h.

During AFM-IR measurements, the AFM tip remained in continuous contact with the surface, enabling simultaneous acquisition of topography and localized infrared absorption. The tunable mid-IR laser was directed onto the sample, and IR absorption induced rapid photothermal expansion. This expansion excited the cantilever’s contact-resonance oscillation, and the resulting oscillation amplitude, proportional to local absorption, was recorded for each pixel, allowing chemical mapping with nanoscale spatial resolution.

A 3 μm × 3 μm region was scanned, and at every pixel the IR laser was tuned to 1615 cm^−1^, corresponding to the aromatic *C* = *C* stretching vibration of the terephthalate ring in PET. This wavenumber was selected because it provides strong PET contrast while minimizing substrate background. A single-frequency chemical map was generated from the cantilever oscillation amplitude at 1615 cm^−1^. Pixels with amplitudes above the baseline noise level, defined from non-absorbing, flat background regions, were automatically highlighted in gold to represent local aromatic absorption.

### 2.4. Thermal characterization of PET bottles.

Differential scanning calorimetry (DSC) measurements were carried out using a TA Instruments Discovery DSC 250 system. Samples of ~ 2–5 mg were extracted from the shoulder of 4 brands of water bottles and placed in an aluminum Tzero DSC pan and lid assembly. Each sample was subjected to two consecutive heating and cooling cycles at 10 °C/min between 40 and 280 °C. For physical aging experiments, the samples were annealed at 60 °C for 12 h in the DSC prior to the two consecutive heating and cooling cycles. All measurements were performed under nitrogen atmosphere.

### 2.5. Survey design and data analysis.

A survey was conducted in Nebraska, USA, as part of the 2023 Nebraska Annual Social Indicators Survey (NASIS). Data collection took place from July 7 to October 15, 2023. Survey packets were mailed to a randomly selected sample of 8000 households using a postal delivery sequence-based sample from the U.S. Postal Service, which ensures broad coverage of approximately 98 % of U.S. households. We received complete responses from 1673 households, which represents a response rate of ~ 21 %. To maintain a probability-based sample, the adult (aged 19 or older) in each household with the next birthday after July 1, 2023, was asked to complete the survey.

The NASIS survey includes two parts: a core set of consistent questions on demographics and quality of life, and a second set of customized questions addressing the specific research needs. In this study, the custom section included questions related to awareness of microplastic contamination in bottled water, total bottled water consumption, and the frequency of bottled water exposure to high-temperature conditions.

To examine associations between socio-economic factors and bottled water usage behaviors, Kendall’s Tau-b correlation analysis was conducted. This non-parametric method, appropriate for ordinal data with tied ranks, assessed the strength and direction of relationships between factors such as population density, household income, education level, and knowledge of microplastics with bottled water consumption and the likelihood of exposing bottled water to heat. Correlation coefficients range from −1 (perfect negative association) to +1 (perfect positive association), and 95 % confidence intervals were calculated using 1000 bootstrapping resamples. To account for multiple comparisons, p-values were adjusted using the Benjamini-Hochberg False Discovery Rate (FDR) procedure. As all significant associations met the FDR threshold of 0.05, unadjusted p-values are reported for ease of interpretation.

## 3. Results and discussion

### 3.1. Quantification of NMP release

#### 3.1.1. Occurrence of NMPs in originally purchased bottled water.

**Fig. 1.**
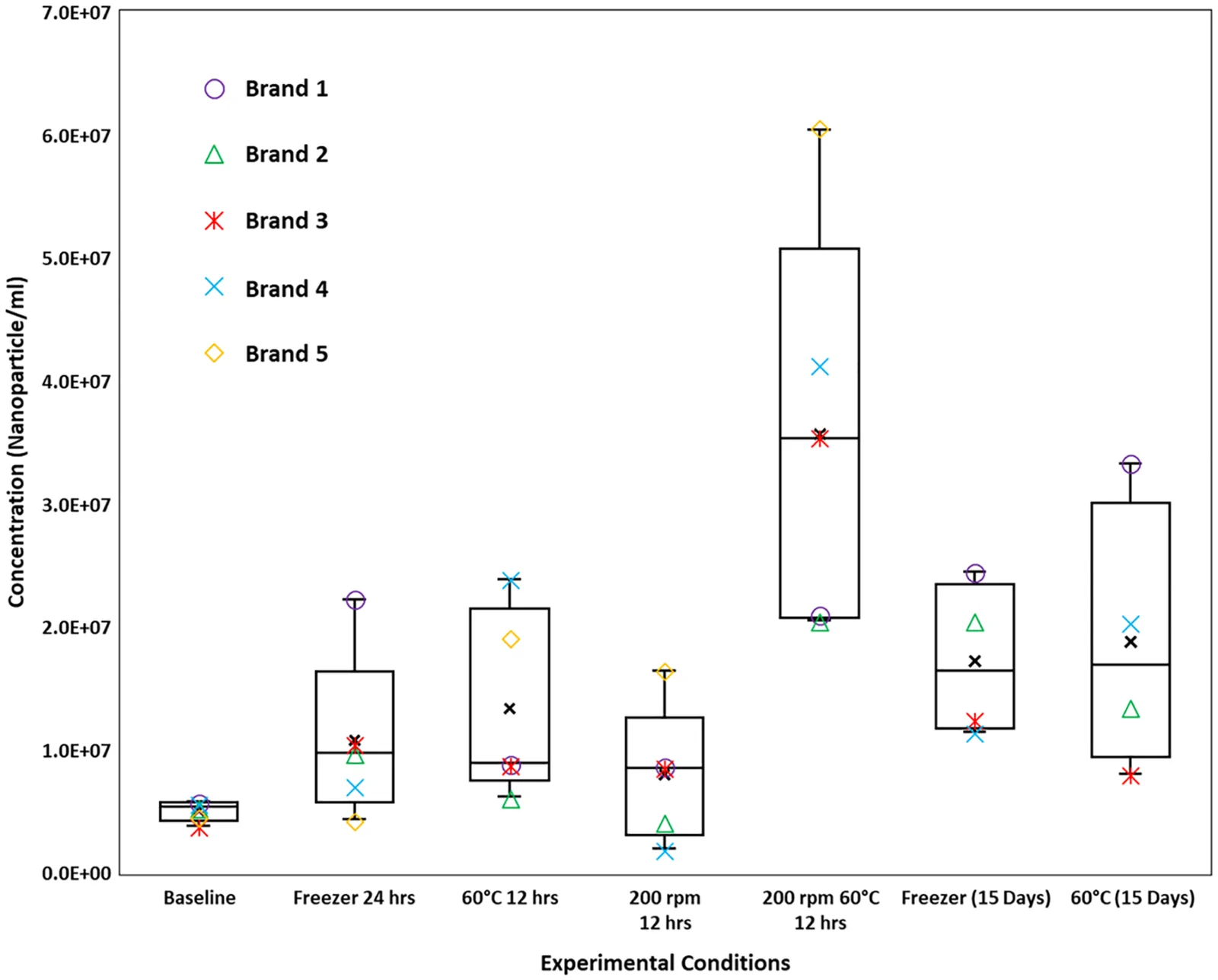
Nanoplastic release from single use PET water bottles under different conditions. Brand 1–5 (N.B: long term high temperature and cold temperature exposure experiments were conducted for only 4 brands).

**Fig. 2.**
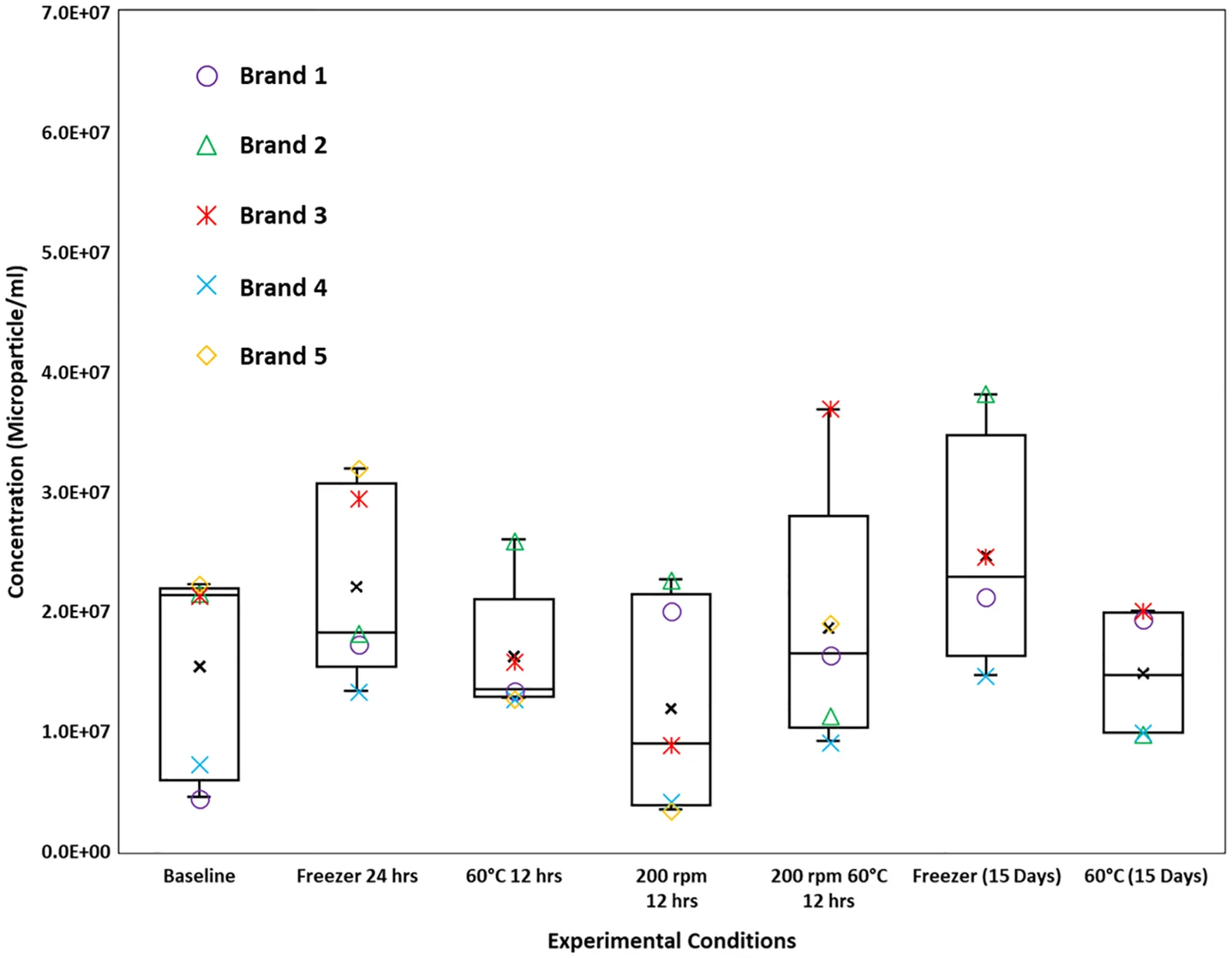
Microplastic release from single use PET water bottles under different conditions. Brand 1–5 (N.B: long term high temperature and cold temperature exposure experiments were conducted for only 4 brands).

A baseline analysis of bottled water from 8 brands, as originally purchased, for NMPs revealed a wide range of particle concentrations. Baseline nanoparticle concentrations spanned from 26.8 million at the high end to 3.81 million per ml at the low end (Figs. 1, S1). Similarly, baseline microparticle concentrations ranged from 23.5 million to 4.49 million per ml (Figs. 2, S2). Experiments using DI water detected 27.6 million nanoparticles and 12.1 million microparticles per ml, comparable to the baseline levels observed in purchased bottled water. Therefore, purchased bottled water was used as the baseline (control), and additional DI water tests were not pursued.

**Fig. 3.**
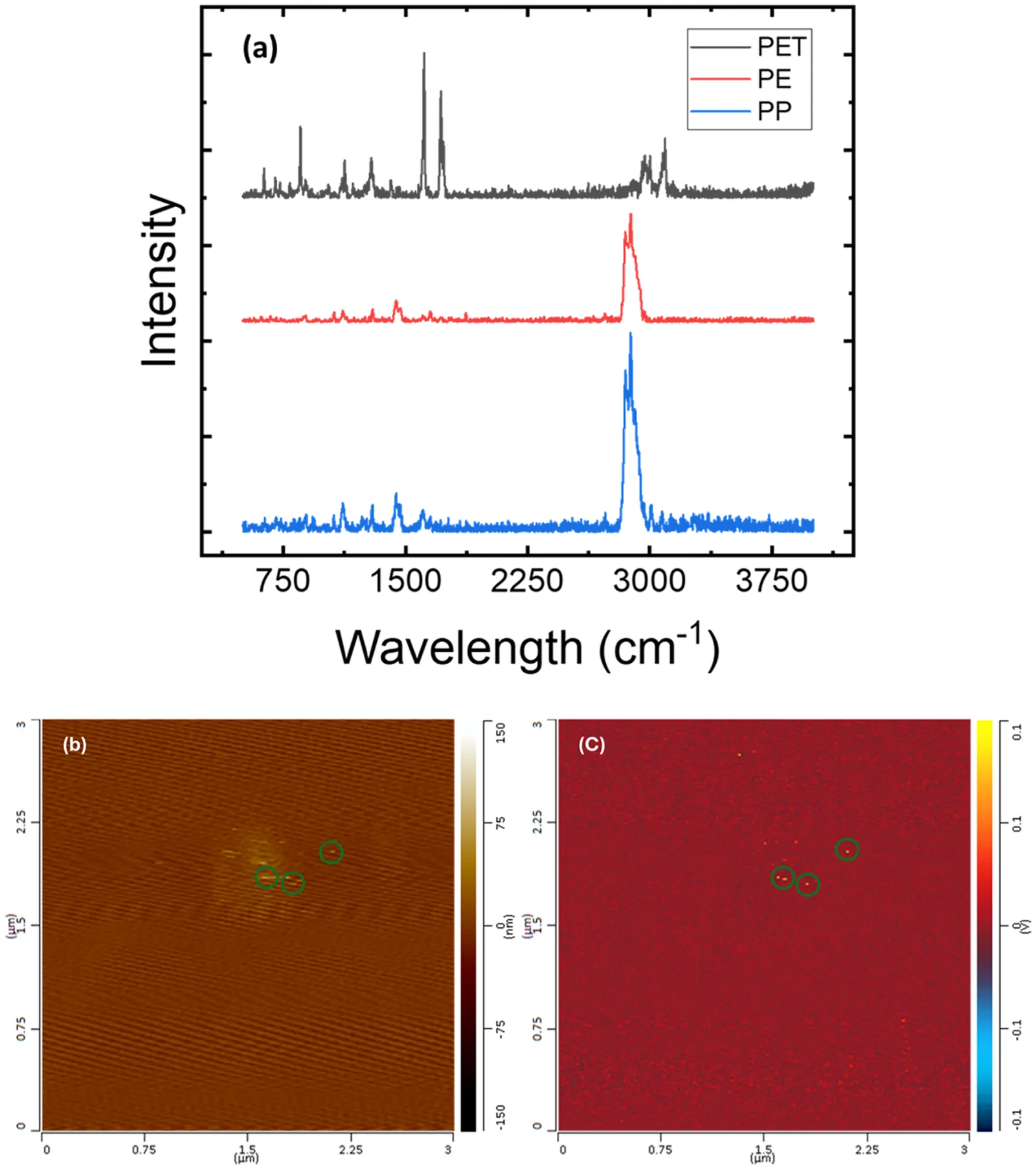
(a) Raman spectra of microplastics analyzed from baseline water sample and water sample collected after the high-temperature shaking experiment. (b, c) AFM topography (b) and nano-IR chemical map (c) collected simultaneously over a 3 μm × 3 μm region. The chemical map shows absorption at 1615 cm^−1^, with gold-highlighted hotspots indicating aromatic polymeric particles consistent with PET nanoplastics released during the experiment.

Notably, brands 6,7, and 8 had baseline particle concentrations approximately 10 times higher than those in brands 1 to 5 (Figs. S1, S2), with the highest baseline concentrations of both nano and microparticles found in brand 6. According to the bottle label, brand 6′s water underwent extensive modifications, including mineral addition and pH balancing. Brand 7 also added minerals after treatment. These treatments likely contributed to the elevated particle counts in brands 6 and 7. Brand 8 was sourced from natural spring water, which might contain natural particles in water. Brands 6, 7, and 8 exhibited substantially higher baseline concentrations, limiting the ability to detect additional particle release unless the increase was exceptionally large. While we conducted all experimental conditions for these brands—except for the temperature cycling scenarios—none resulted in statistically significant changes in particle concentrations. Therefore, no meaningful conclusions can be drawn from these brands. Results for brands 6–8 are provided in the supplemental materials (Figs. S1, S2, Table S2), and the discussion below focuses on brands 1–5, which had lower baseline concentrations and were more responsive to experimental conditions. It is important to note that the reported particle concentrations in our study are significantly higher than those reported in the literature for two primary reasons: 1) our study quantified all particles larger than 10 nm while most previous studies focused on MPs only, and 2) our method includes all types of nano and microparticles (Qian et al., 2024; Zuccarello et al., 2019). As noted below, not all particles detected here are plastic particles (Figs. 3b, 3c, S3, S4).

#### 3.1.2. Impact of cold exposure on particle release.

We evaluated the effects of a single 24-h freezing event on NMP release from eight bottled water brands. Among the five brands with low baseline nanoparticle concentrations (Brands 1–5), mean nanoparticle counts showed a modest increase, but the change was not statistically significant (*t* = 1.95; Table 1). Microplastic concentrations followed a similar pattern, exhibiting no statistically significant change in microparticle concentrations. Freezing-induced expansion of water likely exerts mechanical stress on the bottle interior; however, this alone does not appear sufficient to trigger substantial particle release. These findings are consistent with earlier studies reporting minimal microplastic release from mechanical stress alone in PET bottles (Winkler et al., 2019).

#### 3.1.3. Impact of freeze-thaw cycling on particle release.

During prolonged exposure to freeze-thaw cycles for 15 days, nanoparticle concentrations increased 2.0–4.2 fold across all four brands (Fig. 1). A *t*-test (*p* = 0.026; Table 1) confirmed a statistically significant release of nanoplastics under freeze–thaw cycling. In contrast, microparticle concentrations showed only a modest increase and were not statistically significant (*p* = 0.68).

The likely explanation is repeated mechanical stress from water expansion during freezing. Each cycle may create microfractures or surface wear on the PET interior, gradually releasing embedded particles. Because microparticles are larger and potentially more structurally constrained within the polymer matrix, their release may be less sensitive to such cyclic stress compared to nanoparticles.

#### 3.1.4. Impact of high temperature exposure on particle release.

For nanoparticles, 12 h of exposure at 60 °C led to an increase in nanoparticle concentration for brands 1–5 (Fig. 1), ranging from 1.2 to 4.2 fold above baseline. Statistical analysis (t-statistic = 2.45, p-value = 0.070, Table 1) did not show significance, though a p-value of 0.070 suggest a trend or weak evidence against the null hypothesis, or marginal significance. Thus, the influence of temperature cannot be entirely dismissed, particularly in extreme cases such as brand 4, where an increase of 18.2 million nanoparticles/ml was observed (Fig. 1). For microparticles (Fig. 2), a slight decrease in mean concentrations was observed (t-statistic = −0.34, p-value = 0.748; Table 1), but was not statistically significant.

These results suggest that short-term heating can mobilize nanoparticles, potentially through surface degradation or thermal rearranging of the PET matrix. However, microparticles may require more severe or sustained conditions to be released in measurable amounts.

#### 3.1.5. Impact of high temperature cycling exposure on particle release.

High temperature cycling increased nanoparticle concentrations across all four tested brands: 5.7-, 2.5-, 2.1-, and 3.6-fold for brands 1–4, respectively (Fig. 1). A p-value of 0.075 suggests a trend toward statistical significance, indicating that cycling may accelerate nanoplastic release. Microparticle changes were less consistent (Fig. 2) and not statistically significant (*p* = 0.634).

Over the 15-day experiments, bottles underwent repeated cycles of 12 h at 60 °C followed by 12 h at room temperature, simulating daily heating and cooling. Previous studies have consistently reported an increase in particle release at high temperatures, primarily through thermal degradation and surface flaking (Chen et al., 2023a; Hee et al., 2022; Lee et al., 2024; Zangmeister et al., 2022). However, single-use PET bottles were not tested under dynamic thermal conditions, limiting direct comparisons with our findings.

#### 3.1.6. Impact of high temperature and shaking on particle release.

Among all tested conditions, the combined mechanical shaking at 200 rpm and 60 °C for 12 h produced the greatest particle release. For brands 1–5, shaking at high temperatures resulted in nanoparticle concentrations rising by as much as 9.29 times (Fig. 1), a statistically significant rise (*t* = 4.06, *p* = 0.015; Table 1). Microparticles concentration also increased significantly (*t* = 2.86, *p* = 0.045, Fig. 2), confirming that both particle types are released by the combination of thermal and mechanical stress.

To separate the effects of temperature and agitation, additional experiments were performed on brands 1–5. Shaking alone (200 rpm, 12 h at room temperature) did not significantly change nanoparticle or microparticle levels. Likewise, heating alone (60 °C, 12 h) produced only a weak, non-significant trend of increased nanoparticle concentrations in brands 1–5 (Fig. 1).

Together, these results suggest that neither high temperature nor mechanical agitation alone is sufficient to induce substantial particle release. Instead, it is the combination of both—mimicking extreme conditions with a combination of high temperature and shaking—that leads to the most pronounced release of nano- and microplastic.

### 3.2. Identifications of released particles.

Raman analysis identified polypropylene (PP), PET, and polyethylene (PE) microplastics in the purchased bottled-water (Fig. 3a), with the majority consisting of PP (Fig. 3b). Of the 40 particles analyzed, 53 % were confirmed as PP, 17 % as PET, 5 % as PE, and 25 % remained unidentified. Thus, PP and PET dominated the baseline samples, with PE present in smaller amounts. The unidentified fraction requires further characterization.

In the water samples collected after the combined high temperature and mechanical shaking release experiments, Raman analysis identified PP, PE, and polyethylene terephthalate (PET) (Fig. 3). Of the 40 particles analyzed, 54 % were PET, 28 % were PP, 8 % were PE, and 10 % remained unidentified. SEM–EDX analysis of unidentified particles revealed that the majority are salt crystals, showing strong elemental peaks for ions such as K, Cl, Na, and Ca (Fig. S3). In addition, we detected a single particle with prominent Fe and O signals indicative of iron oxide (Fig. S4). This distribution reflects a clear shift in polymer composition compared with the baseline. Notably, PET rose from 17 % at baseline to 54 % after treatment, while PE increased from 5 % to 8 %. Since the bottle is made of PET and the cap of PE, these results suggest that mechanical stress and elevated temperatures contributed to microplastic release from both the PET bottle and its PE cap. Because only a small subset of particles could be analyzed by Raman spectroscopy, these measurements are intended solely as qualitative confirmation of PET-derived particle release rather than a quantitative representation of the overall particle population.

The simultaneously acquired AFM topography (Figs. 3b, S5, and S6) and nano-IR chemical map (Figs. 3c, S5, and S6) allowed direct correlation between nanoscale morphology and chemical identity. Localized IR-active hotspots centered near 1615 cm^−1^ were observed within the scanned areas. This band is consistent with PET-derived aromatic polymeric material; however, we acknowledge that other aromatic polymers with absorptions in this region cannot be fully excluded based on the current AFM-IR dataset alone. To minimize potential confounding sources, release experiments were conducted using HPLC-grade water and under controlled handling conditions without intentional introduction of other polymer materials. In combination with the known PET-bottle matrix and the presence of a PET-consistent aromatic feature, we interpret these hotspots as PET-derived nanoplastic material, while noting that definitive exclusion of all alternative aromatic polymers would require broader-window nanoscale reference/background spectra.

### 3.3. Thermal aging reveals surface-driven particle release.

**Fig. 4.**
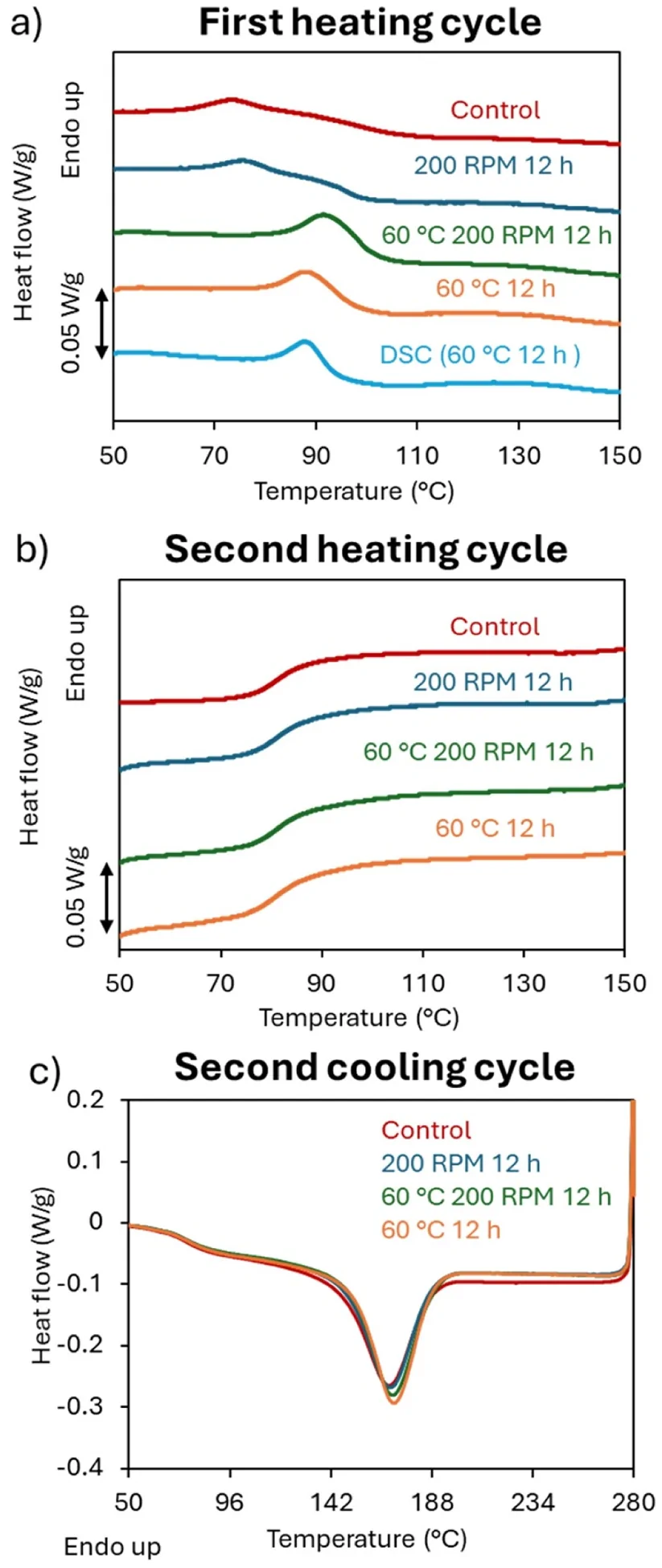
Differential scanning calorimetry of brand 5 PET samples: (a) first heating after bottle treatment and release experiments, (b) second heating, (c) second cooling.

The first DSC heating cycle of PET samples serves to evaluate whether their thermal behavior has been significantly affected by the different heating and agitation treatments that the bottles were subjected to. For brand 5—which showed the most significant changes in particle release for different treatments—the corresponding first DSC heating curves are shown in Fig. 4a and compared to an as-bought control PET sample. An evident enthalpy relaxation peak (~90 °C) appears for all bottle samples that were thermally treated at 60 °C—regardless of whether or not they were subjected to 200 rpm agitation—which is attributed to physical aging of PET (Zhao et al., 2001). Physical aging was confirmed after annealing a control sample at 60 °C for 12 h in the DSC, which resulted in the emergence of the enthalpy relaxation peak at ~90 °C upon subsequent heating. Note that, upon heating, the sample subjected to both heating and agitation conditions (60 °*C* + 200 RPM) shows thermal behavior similar to the sample that was only annealed at 60 °C. Therefore, physical aging cannot explain the dramatic increase in NMP release from bottles that were agitated and heated (60 °*C* + 200 rpm) compared to release from bottles that were only heated (60 °C).

Fig. 4b shows the second DSC heating cycle, where the previous thermal history has been erased. Indeed, no physical aging is present anymore: all DSC curves closely overlap and only exhibit the glass transition of PET ~ 80 °C without an enthalpic relaxation peak. Upon cooling (Fig. 4c), all samples exhibit very similar crystallization peaks, regardless of whether they were subjected to agitation and heating. Given that polymer crystallization is known to be very sensitive to small changes in chain structure, the similarity in crystallization behavior suggests that the different heating and agitation treatments did not result in significant changes or degradation of the bulk polymer. For the other brands, the obtained DSC results were similar to those of brand 5 (Figs. S7–S9).

Overall, these findings suggest that the increased release of particles from bottles exposed to both heat and agitation is unlikely to be caused by bulk structural changes in the plastic. Instead, the release may be driven by surface-level effects, such as abrasion or mechanical stress, rather than by physical aging or by bulk degradation of the PET material. Consistent with the DSC evidence of surface-level physical aging without bulk structural change, SEM imaging revealed only slight surface smoothing and no cracks or large-scale deformation (Fig. S10). This surface smoothing is in line with the PET surface changes observed in a different study(Chen et al. 2023b).

### 3.4. Spatial and socio-behavioral patterns in bottled water use.

#### 3.4.1. Spatial patterns and socio-economic context on NMP exposure from bottled water.

**Fig. 5.**
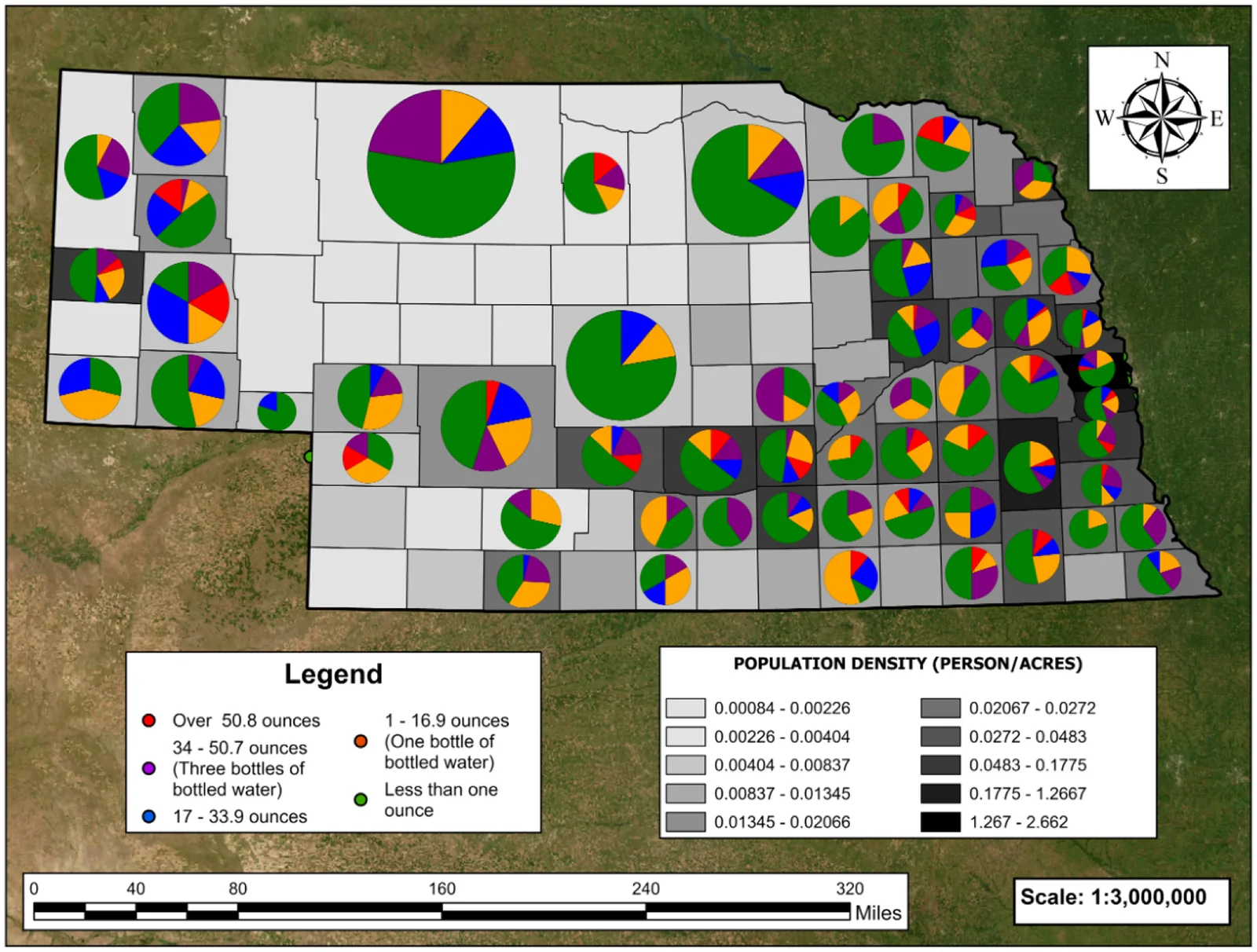
County-level patterns in bottled water use across nebraska. daily bottled water consumption: pie charts show the distribution of consumption levels within each county. background shading reflects population density. (note: counties without a pie chart have fewer than 5 responses.).

Fig. 5 illustrates the geographic distribution of average daily bottled water consumption across Nebraska counties, overlaid on a population density map. Population density is considered a contextual factor since it often aligns with differences in infrastructure, access, and consumption patterns that influence bottled water use and NMP exposure. Each county is represented by a pie chart depicting the proportion of respondents in five consumption categories, from <1 ounce to >50.8 ounces per day. Background shading reflects population density, with darker shades indicating more densely populated areas and lighter shades representing sparser regions. Rural, low-density counties—mainly in western and central Nebraska—tended to have more low-level consumers (<1 oz/day), whereas more densely populated eastern counties showed broader patterns, including higher proportions of moderate to heavy consumers. Although these visual patterns suggest spatial variation in consumption, correlation analysis revealed no significant statistical relationship between population density and bottled water consumption (τ_b_ = −0.0211, Table 2). Similarly, household income showed only a weak, nonsignificant negative association (τ_b_ = −0.0310, Table 2). These findings imply that other factors, such as water quality concerns or cultural preferences, may be more influential than population density or income alone.

#### 3.4.2. Influence of awareness and education on bottled water use and storage behavior.

**Fig. 6.**
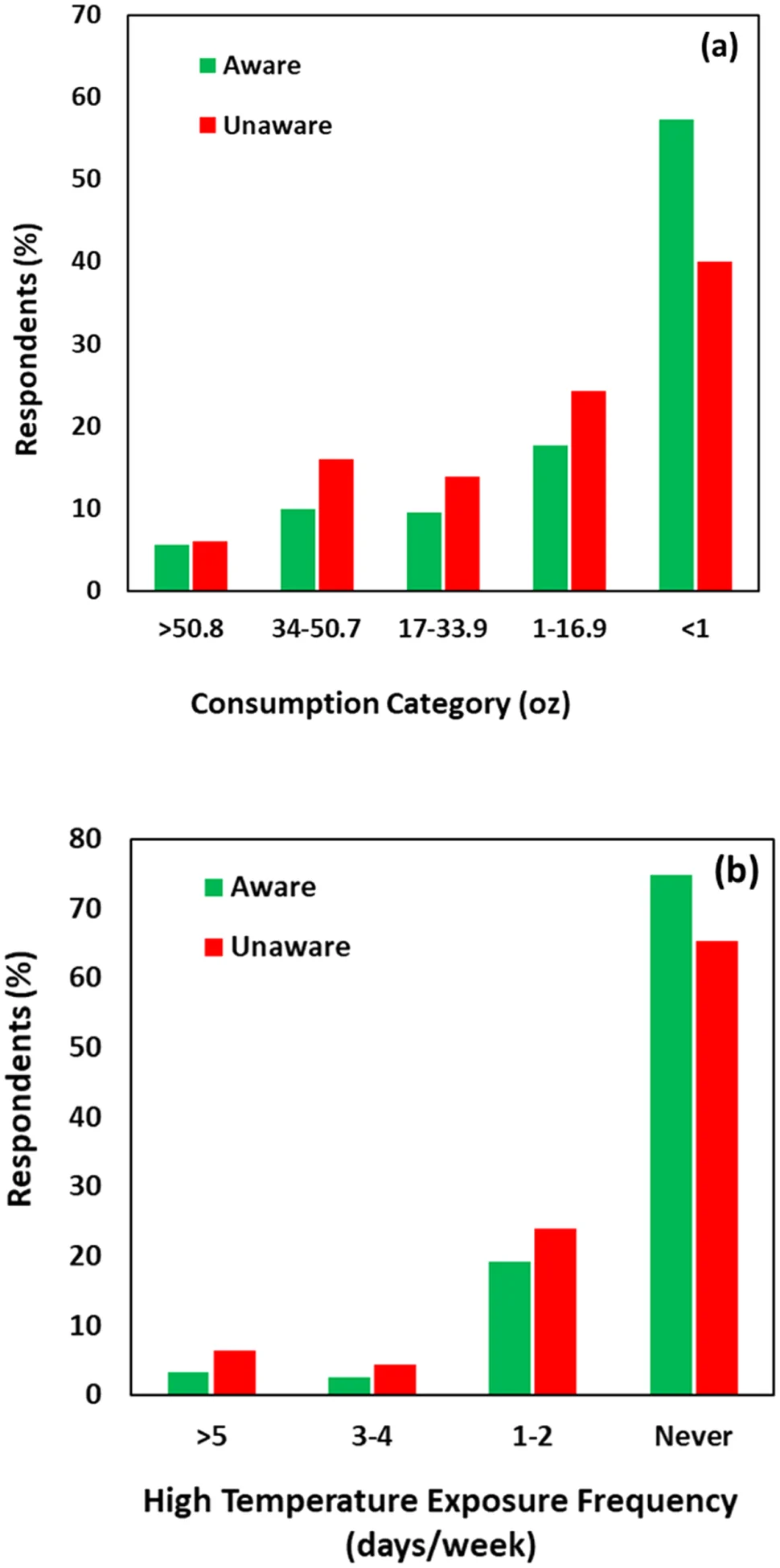
Bottled water consumption and exposure to high temperatures among Nebraska residents. (a) Variation in daily bottled water consumption based on awareness of MPs. (b) Frequency of high-temperature exposure by MP awareness.

Awareness of microplastics and education level showed stronger effects than other socio-economic factors. Over half of the respondents (52.3 %) reported drinking <1 oz of bottled water per day, while only 5.7 % consumed >50.8 oz daily. Individuals who were aware of microplastics were significantly more likely to fall into the lowest consumption category, whereas those unaware of microplastics showed higher consumption across all other categories (Fig. 6). This pattern is reflected in a significant negative Kendall’s Tau-b correlation (τ_b_ = −0.1355, *p* < 0.001, Table 2), indicating that greater awareness is associated with lower bottled water consumption.

Education level was similarly and significantly negatively associated with bottled water use (τ_b_ = −0.1209, *p* < 0.001, Table 2), suggesting that higher educational attainment may also contribute to reduced reliance on bottled water. Together these findings point to awareness and education as stronger predictors of consumption behavior than income and population density, which showed no significant associations.

Patterns of bottled water storage behavior followed a similar trend. While 72 % of respondents reported never exposing bottled water to high temperatures, 28 % did so with varying frequency. Again, individuals aware of microplastics were significantly less likely to expose bottled water to high temperatures (τ_b_ = −0.0992, *p* < 0.001). In contrast, education, income, and population density showed no meaningful association with this behavior (Table 2). These results further underscore the role of microplastic awareness in shaping both consumption and handling practices that influence exposure risk.

## 4. Implications

This study provides the first evidence that routine storage and handling of single-use PET bottled water under real-life conditions can increase human exposure to NMPs. Among the various conditions tested, shaking at elevated temperatures (60 °C with shaking at 200 rpm) produced the greatest release, with nanoparticle concentrations increasing 9.3-fold and microparticles also rising significantly. This underscores the combined role of thermal stress and mechanical forces in degrading PET bottles and caps. Moreover, high-temperature and freeze-thaw cycling over 15 days significantly increased nanoparticle concentrations, particularly in brands with initially low baseline levels. These results emphasize that nano and microplastic release is not limited to extreme scenarios, but can occur under environmentally relevant conditions likely experienced by consumers.

The frequency with which consumers encounter these high-release scenarios depends strongly on everyday storage and handling behaviors. Importantly, survey findings show that individual awareness of microplastics and education level are significantly associated with reduced bottled water consumption and with handling behaviors. Respondents with higher awareness and education reported lower bottled water intake and were less likely to expose bottles to heat—behaviors that may reduce ingestion risk by limiting exposure to the high-release conditions identified in the laboratory experiments. By contrast, income and population density showed only weak associations, suggesting that personal knowledge and habits exert greater influence on exposure risk than broader demographic or economic factors.

Together, these findings indicate that variability in NMP exposure arises not only from differences in particle release under specific conditions, but also from how often different consumer groups experience those conditions. Given these combined insights, several practical mitigation strategies emerge. First, improving public awareness—through educational campaigns or simple guidance on bottle labels advising against storing bottled water in hot environments—may effectively reduce high-risk behaviors that promote particle release. Second, encouraging consumers to avoid leaving bottled water in parked cars, direct sunlight, or other high-temperature settings could significantly lower particle release. Third, promoting alternatives to bottled water in high-heat scenarios, such as reusable bottles made of glass or stainless steel, may further reduce exposure. Finally, stakeholders such as public health agencies, bottled water manufacturers, and retailers could collaborate on clear, consistent messaging that communicates these risks to the public.

By linking mechanistic laboratory findings with real-world behavioral patterns, this study not only identifies key exposure pathways but also highlights feasible, evidence-based actions that can help consumers reduce their potential intake of nano- and microplastics.

## Supplementary Material

1

Supplementary material associated with this article can be found, in the online version, at doi:10.1016/j.watres.2026.125569.

## Figures and Tables

**Table 1:** t-Test results evaluating the statistical significance of effect of different scenarios on nano and microplastics release from brands 1–5.

Experimental Conditions	Nanoplastics		Microplastics	
	t-statistic	p-value	t-statistic	p-value
Freezer 24 hrs	1.95	0.122	−0.73	0.503
60 °C 12 hrs	2.45	0.070	−0.34	0.748
200 rpm 12 hrs	1.08	0.339	−0.54	0.617
200 rpm 60 °C	4.06	0.015	2.86	0.045
Freeze - Thaw (15 Days)	4.10	0.026	0.58	0.600
High Temperature (60 °C)cycling (15 Days)	2.67	0.075	0.53	0.634

**Table 2:** Kendall’s Tau-b correlation coefficients examining the associations between socioeconomic factors, bottled water consumption, and exposure to high temperatures.

Variables	Bottles water consumption			Exposure to high temperature		
	τ_b_	95 % CI	p-value	τ_b_	95 % CI	p-value
Population density	−0.0212	[−0.0559, 0.0136]	0.232	−0.0014	[−0.0397, 0.0368]	0.941
Household income	−0.0332	[−0.0740, 0.0076]	0.111	0.0115	[−0.0276, 0.0506]	0.565
Education level	−0.1209	[−0.1605, −0.0813]	<0.001	0.0219	[−0.0210, 0.0648]	0.316
Knowledge of MPs	−0.1355	[−0.1801, −0.0909]	<0.001	−0.0992	[−0.1470, −0.0514]	<0.001

## Data Availability

Data will be made available on request.

## HIGHLIGHTS

- PET bottles release nano/microplastics under realistic storage and handling.
- Heat plus shaking boosted nanoplastics 9× and microparticles significantly.
- Freeze–thaw and heat cycling consistently elevated nanoplastic release.
- Survey: awareness and education reduced bottled water use and heat exposure.
