# Supplementary material for: Everyday storage and handling of PET bottled water increase human exposure to nano- and microplastics: Influence of socio-economic factors
Source: Water Res. Author manuscript; Available in PMC 2026 Sep 4. (PMC13543205; doi:10.1016/j.watres.2026.125569)
Supplement: Supplementary file 1 [file NIHMS2178248-supplement-1.docx]

**Quantification of Nano and Microplastic Release from Single-Use PET Bottles under Real-Life Conditions: Assessing the Influence of Socio-Economic Factors on Human Exposure**

In preparation for

Water Research

January 2026

Kazi Albab Hussain^a^, Haoyu Dong^b^, Alex Tamayo^c^, Seulki Kim^d^, Cesar Augusto Gomez Pena^a^, Silvia Foss^e^, Lucia Fernandez-Ballester^c^, Yongfeng Lu^b^,and Yusong Li^*a^

^a^Department of Civil and Environmental Engineering, University of Nebraska-Lincoln, Lincoln, Nebraska 68588-053, United States

^b^Department of Electrical & Computer Engineering, University of Nebraska─Lincoln, Lincoln, Nebraska 68588-0511, United States

^c^Department of Mechanical & Materials Engineering, University of Nebraska-Lincoln, Lincoln, Nebraska 68588-0526, United States

^d^Department of Sociology, University of Nebraska-Lincoln, Lincoln, Nebraska 68588, United States

^e^Lincoln High School, Lincoln, NE 68510, United States

**Corresponding Author**

Yusong Li − Department of Civil and Environmental Engineering, University of Nebraska-Lincoln, Lincoln, Nebraska 68588-053, United States

Email: yli7@unl.edu

**Text S1:** Operational Steps for Nanoparticle Tracking Analysis (NTA.

**Instrument Preparation.** Before each measurement session, we powered on the NTA instrument and allowed the laser and camera system to warm up for approximately 20–30 minutes. The sample chamber and tubing were flushed several times with HPLC-grade water to remove any residual particles. This process was repeated until the flow channel was confirmed to be clean, as indicated by the absence of detectable particles when running HPLC-grade water as a blank.

**Sample Preparation.** All samples were mixed gently by inversion to avoid air-bubble formation. Measurements were initiated with the sample expected to have the lowest particle concentration to minimize the possibility of carryover. No vortexing or harsh agitation was used at any stage.

**Sample Loading.** Each sample was introduced into the analysis chamber using a 1-mL syringe and loaded slowly using the syringe pump to prevent bubble formation. A low and constant flow rate was applied to maintain stable particle movement throughout each measurement, and the same flow rate was used for all samples.

**Camera Alignment and Parameter Settings.** The microscope focus was adjusted until particles appeared as sharp, high-contrast points. The camera level was held constant at 14 for all measurements, and the detection threshold was fixed at 5 across the entire dataset to ensure consistency. These settings were optimized during initial calibration and maintained throughout the experiment.

**Video Capture.** For each sample, three videos were acquired, each with a duration of 60 seconds. All capture parameters—including camera level, detection threshold, and flow rate—were kept constant across all samples and replicates to maintain comparability.

**Data Analysis.** Video files were processed using the NTA software to track the Brownian motion of particles and calculate hydrodynamic diameters via the Stokes–Einstein equation. A fixed detection threshold was applied for all samples within each experimental batch. The resulting outputs included particle size distributions and particle concentrations (particles/mL), which were exported for subsequent statistical analysis.

**Quality Control and Cleaning Between Samples.** After each measurement, the analysis chamber was thoroughly rinsed with HPLC-grade water until the particle signal returned to baseline. Blank measurements were periodically performed to confirm the absence of carryover. At the end of each measurement session, the chamber was cleaned by flushing with HPLC-grade water followed by ethanol or an instrument-recommended cleaning solution.

**Text S2:** **Salt spike–recovery experiment.** To evaluate the influence of dissolved salts and assess the robustness of the baseline subtraction procedure, a salt spike–recovery experiment was conducted by adding 0.9% NaCl to deionized water in three replicates and analyzing the samples using the same workflow as the control. For the control (DI water), the mean particle concentration was 2.84 × 10⁶ ± 2.23 × 10⁶ particles mL⁻¹. After salt addition, the measured concentration increased to 5.43 × 10⁶ ± 2.73 × 10⁶ particles mL⁻¹. The increase observed following the 0.9% salt spike indicates that the baseline subtraction approach adequately accounts for salt-related background contributions, supporting the robustness of the measurement method.

**Table S1:** Conductivity of bottled water brands analyzed in this study.

| **Brand** | **Conductivity (Mean ± SD) (µs/cm)** |
| --- | --- |
| Brand 1 | 2.50 ± 0.26 |
| Brand 2 | 58.00 ± 0.20 |
| Brand 3 | 55.13 ± 0.21 |
| Brand 4 | 3.17 ± 0.31 |
| Brand 5 | 63.77 ± 0.15 |
| Brand 6 | 52.40 ± 0.10 |
| Brand 7 | 46.60 ± 0.26 |
| Brand 8 | 103.83 ± 0.47 |

**FIGURE S1.** Nanoplastic release from single use PET water bottles under different conditions; brand 6-8.

**FIGURE S2.** Microplastic release from single use PET water bottles under different conditions; brand 6-8.

**Table S2:** t-Test Results Evaluating the Statistical Significance of Effect of Different Scenarios on Nano and Microplastics Release from Brands 6-8

| Experimental Conditions | Nanoplastics | | Microplastics | |
| --- | --- | --- | --- | --- |
|  | t-statistic | p-value | t-statistic | p-value |
| Freezer 24 hrs | -0.62 | 0.597 | -0.23 | 0.836 |
| 60°C 12 hrs | 0.47 | 0.683 | -0.38 | 0.739 |
| 200 rpm 60°C | 1.38 | 0.301 | -5.54 | 0.031 |

**FIGURE S3.** SEM–EDX characterization of a representative non-plastic particle. The particle exhibits strong elemental peaks for potassium (K) and chloride (Cl), along with weaker signals for aluminum (Al) and magnesium (Mg).

**FIGURE S4.** SEM–EDX characterization of a representative non-plastic particle. The particle exhibits strong elemental maps of iron and oxygen (b-d), consistent with iron oxide.

Figure S5. AFM height image (left) and corresponding AFM-IR chemical map (right).

.

Figure S6. AFM height image (left) and corresponding AFM-IR chemical map (right) of the sample surface. Individual particles are not clearly resolved in the AFM height image due to reduced topographic contrast, likely caused by salt accumulation on the tip during contact-mode operation; however, localized IR-active hotspots are evident in the chemical map.

.

**FIGURE S7.** DSC analysis of brand 1.

**FIGURE S8.** DSC analysis of brand 4.

**FIGURE S9.** DSC analysis of brand 6.

**Figure S10.** Scanning electron microscopy (SEM) images of the PET bottle inner surface before and after exposure. (a) New, unused bottle showing the characteristic PET morphology. (b) Bottle exposed to 60 °C with 200 rpm shaking, displaying slight surface smoothing but no visible cracks, fractures, or severe mechanical wear.

**Notes**

The authors declare no competing financial interest.

**ACKNOWLEDGMENTS**

This research was supported by the University of Nebraska-Lincoln Grand Challenge Catalyst Project titled Release of Micro- and Nanoplastics from Plastic Food Containers: Characterization, Risk Assessment, Socioeconomic, and Health Impacts.
